# Advancing glaucoma detection with convolutional neural networks: a paradigm shift in ophthalmology

**DOI:** 10.22336/rjo.2023.39

**Published:** 2023

**Authors:** Shafeeq Ahmed Haja, Vidyadevi Mahadevappa

**Affiliations:** * Department of Ophthalmology, Bangalore Medical College and Research Institute, India

**Keywords:** glaucoma, artificial intelligence, fundoscopy, Convolutional Neural Networks, retina

## Abstract

A leading cause of irreversible vision loss, glaucoma needs early detection for effective management. Intraocular Pressure (IOP) is a significant risk factor for glaucoma. Convolutional Neural Networks (CNN) demonstrate exceptional capabilities in analyzing retinal fundus images, a non-invasive and cost-effective imaging technique widely used in glaucoma diagnosis. By learning from large datasets of annotated images, CNN can identify subtle changes in the optic nerve head and retinal structures indicative of glaucoma. This enables early and precise glaucoma diagnosis, empowering clinicians to implement timely interventions. CNNs excel in analyzing complex medical images, detecting subtle changes indicative of glaucoma with high precision. Another valuable diagnostic tool for glaucoma evaluation, Optical Coherence Tomography (OCT), provides high-resolution cross-sectional images of the retina. CNN can effectively analyze OCT scans and extract meaningful features, facilitating the identification of structural abnormalities associated with glaucoma. Visual field testing, performed using devices like the Humphrey Field Analyzer, is crucial for assessing functional vision loss in glaucoma. The integration of CNN with retinal fundus images, OCT scans, visual field testing, and IOP measurements represents a transformative approach to glaucoma detection. These advanced technologies have the potential to revolutionize ophthalmology by enabling early detection, personalized management, and improved patient outcomes. CNNs facilitate remote expert opinions and enhance treatment monitoring. Overcoming challenges such as data scarcity and interpretability can optimize CNN utilization in glaucoma diagnosis. Measuring retinal nerve fiber layer thickness as a diagnostic marker proves valuable. CNN implementation reduces healthcare costs and improves access to quality eye care. Future research should focus on optimizing architectures and incorporating novel biomarkers. CNN integration in glaucoma detection revolutionizes ophthalmology, improving patient outcomes and access to care. This review paves the way for innovative CNN-based glaucoma detection methods.

**Abbreviations: **CNN = Convolutional Neural Networks, AI = Artificial Intelligence, IOP = Intraocular Pressure, OCT = Optical Coherence Tomography, CLSO = Confocal Scanning Laser Ophthalmoscopy, AUC-ROC = Area Under the Receiver Operating Characteristic Curve, RNFL = Retinal Nerve Fiber Layer, RNN = Recurrent Neural Networks, VF = Visual Field, AP = Average Precision, MD = Mean Defect, sLV = square-root of Loss Variance, NN = Neural Network, WHO = World Health Organization

## Introduction

Glaucoma is a group of eye diseases that cause damage to the optic nerve, resulting in progressive and irreversible vision loss. It is one of the leading causes of blindness worldwide, estimated to have affected approximately 112 million people by 2020 [**1**]. Glaucoma is often asymptomatic in the early stages, making early detection and diagnosis crucial for timely treatment. The impact of glaucoma on vision can range from peripheral vision loss to complete blindness, severely affecting a person’s quality of life and independence [**2**,**3**].

Convolutional Neural Networks (CNN) have revolutionized the field of medical image analysis by providing a powerful and effective approach to processing visual data. Unlike traditional machine learning algorithms, CNNs can automatically learn and extract relevant features directly from the input images, eliminating the need for manual feature engineering [**4**]. This capability makes CNN well-suited for complex and high-dimensional data such as medical images. One of the key strengths of CNNs is their hierarchical architecture, which consists of multiple layers of interconnected neurons. Each layer performs a specific function, such as feature extraction or classification, and the information flows through the network in a feed-forward manner [**5**,**6**].

The core building block of a CNN is the convolutional layer, which applies a set of learnable filters to the input image, capturing local patterns and spatial relationships. These filters enable the network to automatically detect edges, corners, textures, and other image features relevant to the task at hand [**7**]. The ability of CNNs to learn directly from data is particularly advantageous in medical image analysis, in which the interpretation and extraction of meaningful features can be challenging. For example, in glaucoma diagnosis, CNNs can learn to detect subtle changes in the optic nerve head and retinal structures that are indicative of the disease [**8**]. By training the network on large datasets of annotated images, CNN can learn to generalize and accurately classify new unseen images, enabling automated and efficient diagnosis.

**Fig. 1:**
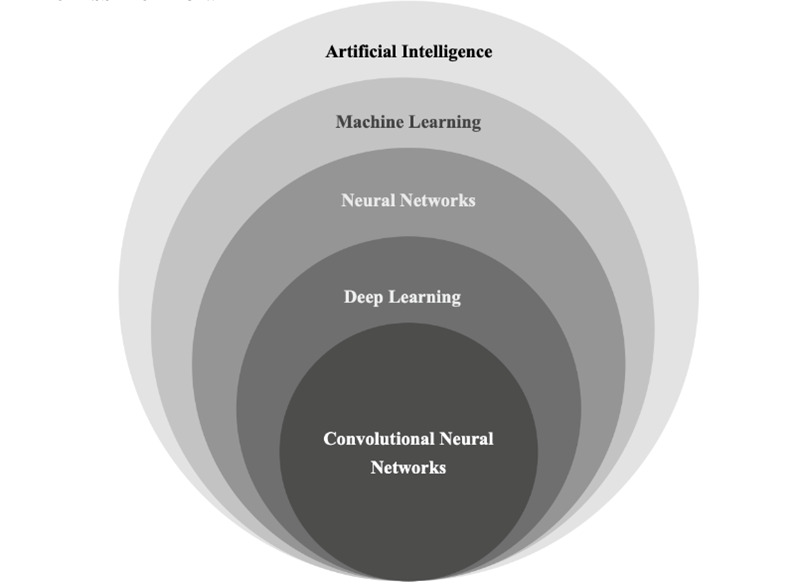
AI classification

In the field of medical image analysis, CNNs have proven to be a groundbreaking technology with remarkable capabilities. CNNs excel in various tasks, including image segmentation, object detection, and disease classification [**9**]. By leveraging deep learning techniques, CNN can learn from extensive datasets and autonomously extract relevant features from medical images. This unique ability empowers them to identify subtle patterns and anomalies that may go unnoticed by human observers [**10**]. As a result, CNNs have opened up new possibilities for automated disease diagnosis and treatment planning. The strength of CNNs lies in their capacity to process large amounts of visual data and uncover intricate relationships within the images [**11**]. Through a hierarchical architecture of interconnected layers, CNN can effectively capture both low-level and high-level features, allowing them to discern complex structures and patterns in medical images. **Fig. 1** shows a representation of different Artificial Intelligence (AI) sub-classifications. This deep understanding of image content enables CNN to perform tasks like image segmentation, where they can accurately delineate different anatomical structures or regions of interest within an image.

## Fundamentals of glaucoma diagnosis

*Overview of traditional diagnostic methods for glaucoma*

Traditional diagnostic methods for glaucoma involve several key components to assess the condition. The first important aspect is the measurement of intraocular pressure (IOP), as elevated IOP is a major risk factor for glaucoma. By using techniques such as tonometry, healthcare professionals can obtain an accurate measurement of IOP [**12**]. Elevated IOP can lead to damage to the optic nerve and subsequent vision loss, making its measurement crucial in glaucoma diagnosis. Visual field testing is another essential component of glaucoma diagnosis [**13**,**14**]. Commonly performed using perimetry, this test evaluates the patient’s peripheral and central vision. It helps detect any visual field defects or abnormalities associated with glaucoma [**15**]. By mapping out the patient’s field of vision, healthcare professionals can identify areas of vision loss or impaired sensitivity, providing valuable information about the progression and severity of the disease [**16**].

Examination of the optic nerve is a critical step in glaucoma diagnosis. This involves assessing the cup-to-disc ratio, which measures the proportion of the optic disc occupied by the cup. An increased cup-to-disc ratio can indicate optic nerve damage and is often associated with glaucoma [**17**]. Evaluating the appearance of the optic disc and looking for other signs of optic nerve damage, such as nerve fiber layer thinning or hemorrhages, further aids in diagnosing the condition [**18**].

*Limitations of conventional diagnostic techniques*

While traditional diagnostic methods for glaucoma have been the standard for many years, they do have certain limitations. One of the primary limitations is their reliance on subjective interpretation, which can introduce variability and potential human error into the diagnostic process. For example, visual field testing can be subjective and may not detect early or subtle visual field defects. This can lead to delayed diagnosis and treatment, potentially resulting in further vision loss.

Additionally, traditional diagnostic techniques may not provide a complete picture of the disease [**19**-**21**]. Relying solely on intraocular pressure (IOP) measurements, which have been a cornerstone of glaucoma diagnosis, may not be sufficient as some individuals with normal IOP can still develop glaucoma. This highlights the need for additional objective measures to complement IOP measurements and improve diagnostic accuracy. Moreover, these methods may not be suitable for accurate diagnosis in certain populations, such as patients with complex eye conditions or those with limited cooperation or understanding. In such cases, alternative diagnostic approaches are needed to overcome these challenges and ensure accurate diagnosis and timely intervention [**22**,**23**].

The introduction of fundus imaging has addressed many of these limitations and revolutionized glaucoma diagnosis. Fundus imaging techniques, such as fundoscopy, optical coherence tomography (OCT), and confocal scanning laser ophthalmoscopy (CSLO), provide objective and quantitative data, enabling precise measurements of the optic nerve morphology and retinal structure [**20**]. This allows for the early detection of glaucomatous damage, even before significant visual field defects are observed. Additionally, the quantitative nature of the data obtained from fundus imaging reduces the reliance on subjective interpretation, improving diagnostic accuracy and consistency.

*Introduction to fundus imaging and its role in glaucoma diagnosis*

**Fig. 2:**
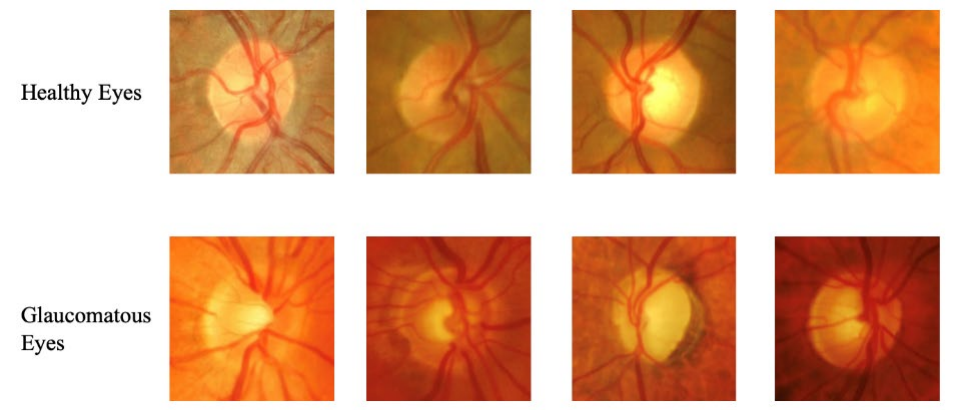
Fundus images of healthy eyes and glaucomatous eyes localized at the optic nerve head

Fundus imaging, specifically imaging of the optic nerve head and the surrounding retinal tissue, has emerged as a valuable and non-invasive tool in glaucoma diagnosis. This advanced imaging technique allows for detailed visualization of the optic disc and enables the assessment of structural changes associated with glaucoma. By capturing high-resolution images of the retina, optic nerve head, and nerve fiber layer, fundus imaging provides objective measurements and quantitative data for analysis, enhancing diagnostic accuracy and enabling precise monitoring of disease progression [**24**]. **Fig. 2** shows the different variations in fundus images of healthy versus glaucomatous patients. One of the key advantages of fundus imaging is its ability to visualize subtle changes in the optic disc, such as optic disc cupping, neuroretinal rim thinning, and retinal nerve fiber layer defects. These changes are crucial indicators of glaucoma progression and can provide valuable insights into the severity and stage of the disease. Fundus imaging allows for the precise measurement of important parameters, such as cup-to-disc ratio, rim thickness, and retinal nerve fiber layer thickness, which are essential for monitoring glaucoma over time [**25**].

Furthermore, fundus imaging offers the advantage of objective and quantitative assessment, reducing the reliance on subjective interpretation and inter-observer variability. The captured images can be analyzed using specialized software algorithms, allowing for precise measurements and automated detection of glaucomatous changes. This not only improves diagnostic accuracy but also enhances the consistency and reproducibility of the results [**26**]. In addition to the diagnosis, fundus imaging plays a crucial role in the management of glaucoma. Serial fundus images can be compared over time to assess the progression of the disease and evaluate the effectiveness of treatment interventions. This longitudinal monitoring enables timely adjustments in the treatment plan and facilitates early intervention to prevent further vision loss [**27**].

*Importance of accurate and timely glaucoma detection*

Accurate and timely detection of glaucoma is of paramount importance in preserving patients’ vision and preventing further damage. Early detection allows for interventions and treatments to be initiated at a stage when vision loss can still be minimized. By implementing advanced imaging techniques like fundus imaging, healthcare professionals can improve the accuracy and sensitivity of glaucoma diagnosis [**28**]. Fundus imaging provides objective and quantitative measurements, enabling better tracking of disease progression over time. It allows for the precise assessment of the structural changes in the optic disc and retinal tissue, providing valuable insights into the severity and stage of glaucoma. These detailed images help in identifying subtle signs of glaucomatous damage, such as optic disc cupping, neuroretinal rim thinning, and retinal nerve fiber layer defects [**29**].

In addition to aiding in the diagnosis, fundus imaging assists in identifying individuals at risk for developing glaucoma. It can detect pre-glaucomatous conditions or high-risk characteristics, enabling proactive management and timely intervention. Early detection of glaucoma-related changes in the fundus can prompt healthcare professionals to closely monitor these individuals and implement preventive measures to preserve their vision [**30**]. Moreover, accurate glaucoma detection is crucial for implementing appropriate treatment strategies. Based on the findings from fundus imaging, healthcare professionals can determine the most suitable management options for each patient. This may include medical therapy, laser treatment, or surgical interventions, tailored to the specific needs of the individual. Early diagnosis allows for the monitoring of treatment efficacy and disease progression, facilitating timely adjustments in the treatment plans as required [**31**,**32**].

## CNN in glaucoma diagnosis

*CNN architecture and its suitability for image classification*

CNNs have emerged as a powerful tool for image classification tasks, including glaucoma diagnosis. The architecture of a CNN, with its distinctive layers and operations, is well-suited for handling the complexity of medical image analysis. The suitability of CNN for image classification is due to the following reasons:

1. Convolutional Layers. These layers apply filters to the input image, enabling the network to learn local patterns and features. The filters detect edges, textures, and other relevant characteristics automatically. By capturing these features at different spatial scales, CNN can identify meaningful structures within the images [**33**].

2. Pooling Layers. Pooling layers downsample the feature maps produced by the convolutional layers. They reduce the spatial dimensions while preserving the essential information. Pooling helps in extracting the most relevant features and making the network more robust to variations in position and scale [**34**].

3. Fully Connected Layers. These layers aggregate the learned features and make predictions based on them. The fully connected layers combine the spatial information captured by the previous layers and transform it into a format suitable for classification. They provide the network with the ability to reason about global relationships and make high-level decisions [**33**].

The hierarchical nature of CNN architecture makes it highly suitable for image classification tasks. CNN can learn hierarchical representations of the input images, starting from low-level features like edges and textures, and gradually progressing to more abstract and complex features. This hierarchical representation allows CNN to distinguish between different classes of images effectively [**35**]. In the case of glaucoma diagnosis, accurate classification of medical images is crucial. With their ability to learn and extract relevant features automatically, CNN can effectively capture the subtle patterns and abnormalities associated with glaucoma [**36**]. By analyzing the raw pixel data, CNN can identify distinct features indicative of glaucoma, such as optic disc cupping, neuroretinal rim thinning, and retinal nerve fiber layer defects.

*CNN training and validation*

Training a CNN for glaucoma diagnosis involves two key processes: training and validation. These processes play a crucial role in developing and evaluating CNN’s performance.

**Fig. 3:**
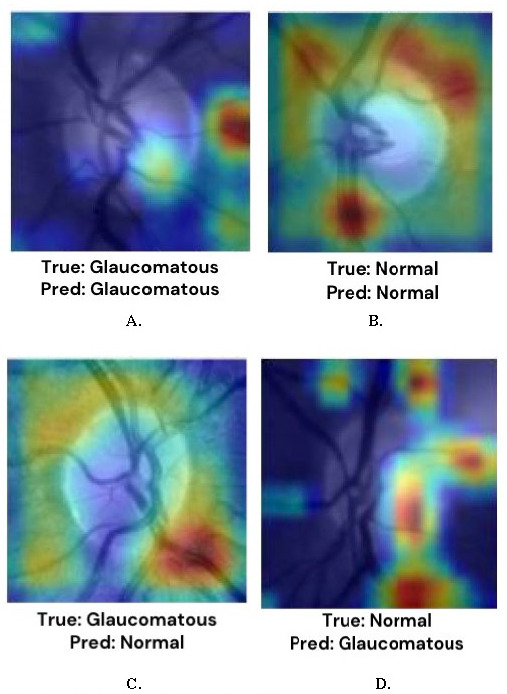
Regional sensitivity for model prediction of glaucomatous patients from fundus imaging. **(A)** The model’s prediction aligns with the ground truth diagnosis, indicating that it accurately classified the input data as belonging to the glaucomatous category. **(B)** The model’s prediction aligns with the ground truth diagnosis, indicating that it accurately classified the input data as belonging to the normal category. **(C)** This is a false negative result, where the model failed to detect the presence of glaucoma in the sample. **(D)** This is a false positive result, where the model failed to detect the presence of normal fundus in the sample

The training process begins by providing the CNN with a large dataset of labeled images. Each image is associated with a specific class, such as glaucoma-positive or glaucoma-negative. Through a technique called backpropagation, the CNN learns from these labeled images and adjusts its internal parameters, including weights and biases. Backpropagation calculates the gradient of the loss function concerning the CNN’s parameters, enabling the network to update its parameters in a way that minimizes prediction errors [**37**]. During the training process, CNN gradually improves its ability to recognize patterns and features that are indicative of different classes of images. It learns to differentiate between various image characteristics that are associated with glaucoma or non-glaucoma conditions. By iteratively adjusting its parameters, CNN becomes more proficient at making accurate predictions. Once the CNN is trained, it undergoes a validation process to evaluate its performance. A separate validation dataset, consisting of images that were not used during training, is employed for this purpose. The CNN makes predictions on these unseen images, and various metrics, such as accuracy, are computed to assess its performance [**33**]. **Fig. 3** shows the model recognition areas in a fundus image along with the associated response.

The validation process serves several important functions. Firstly, it helps in monitoring CNN’s generalization ability. Generalization refers to the network’s capacity to accurately classify new, unseen images. By evaluating CNN’s performance on the validation dataset, we can determine how well it can generalize its learned knowledge to make accurate predictions on new cases. Moreover, the validation process allows for the identification of potential issues such as overfitting [**38**]. Overfitting occurs when a CNN becomes too specialized to the training dataset and fails to generalize well to new data. By evaluating CNN’s performance on unseen images, we can assess whether it has learned meaningful representations that can be applied to new cases. The accuracy and other evaluation metrics obtained during the validation process provide valuable insights into CNN’s performance. They serve as indicators of the network’s effectiveness in classifying glaucoma-related features in medical images [**39**]. This information is crucial for assessing the CNN’s readiness for real-world deployment in glaucoma diagnosis and management.

*Various CNN models used in glaucoma diagnosis*

Several CNN models have been utilized in glaucoma diagnosis, each with its own unique architecture and performance characteristics. Among the popular CNN models used in this field are VGG-16, ResNet, and Inception v3. 

VGG-16 is a deep CNN architecture that consists of 16 layers, including multiple convolutional layers with small-sized filters. It gained prominence for its exceptional performance in image classification tasks, including glaucoma detection. VGG-16 has demonstrated the ability to learn and extract meaningful features from glaucoma images, leading to accurate classification results [**40**,**41**]. 

ResNet, short for Residual Network, introduced the concept of residual connections. These connections allow the network to skip certain layers, which facilitates the training of much deeper networks. By addressing the problem of vanishing gradients, ResNet enables the successful training of CNN with a large number of layers. In the context of glaucoma diagnosis, ResNet has shown improved accuracy and the ability to capture more intricate features in glaucoma images, leading to enhanced diagnostic performance [**4**].

Inception v3, also known as GoogLeNet, introduced the concept of “inception modules”. These modules consist of parallel convolutional operations with different filter sizes, allowing the network to capture features at multiple scales. By concatenating the outputs of these parallel operations, Inception v3 enhances the network’s ability to learn discriminative features. In glaucoma diagnosis, Inception v3 has exhibited strong performance, achieving high accuracy in classifying glaucomatous and healthy images [**41**].

Among others, these CNN models have been successfully applied in glaucoma diagnosis due to their ability to learn and extract meaningful features from medical images. By leveraging their deep architectures and sophisticated operations, these models can capture both low-level and high-level features, enabling accurate classification of glaucoma-related patterns. Moreover, these models can handle the inherent complexity and variability present in glaucoma images, making them robust tools for assisting healthcare professionals in diagnosing and managing the disease [**4**].

*The power of CNN in achieving unparalleled accuracy and specificity*

CNNs have revolutionized glaucoma detection by significantly enhancing the accuracy and specificity of the diagnostic process. Despite being valuable, traditional diagnostic methods for glaucoma are often limited by subjective interpretations and the potential to overlook early or subtle signs of the disease [**42**]. However, with their remarkable ability to automatically learn relevant features from vast amounts of data, CNNs have emerged as a game-changer in the field. 

The fundamental strength of CNNs lies in their capacity to extract and analyze intricate patterns and abnormalities within medical images, including those associated with glaucoma. By learning from large datasets, CNN can capture subtle variations that might elude human observers. This leads to more accurate and reliable detection of glaucoma, enabling timely interventions and preventing further vision loss [**37**]. The exceptional accuracy and specificity achieved by CNN in glaucoma detection have far-reaching implications for patient care. Through their automated feature extraction capabilities, CNNs excel at capturing both low-level and high-level features from medical images. This comprehensive understanding of visual information enables CNN to differentiate between healthy and glaucomatous conditions with unprecedented accuracy [**32**].

By leveraging CNN, healthcare professionals can achieve early and precise glaucoma diagnosis, allowing for timely interventions tailored to each patient’s needs. Early detection empowers clinicians to implement appropriate treatment strategies, whether medical therapy, laser treatment, or surgical interventions [**40**]. Additionally, CNNs enable the monitoring of treatment efficacy and disease progression, facilitating timely adjustments in treatment plans as required. Furthermore, CNNs exhibit exceptional specificity, reducing the risk of false-positive or false-negative diagnoses. The ability to accurately differentiate between normal and glaucomatous conditions ensures that patients receive appropriate care based on their individual needs. This specificity not only optimizes patient outcomes but also conserves valuable healthcare resources by minimizing unnecessary interventions or missed diagnoses [**15**].

The impact of CNN in achieving high accuracy and specificity in glaucoma detection extends beyond the confines of traditional diagnostic techniques. These advanced algorithms provide healthcare professionals with invaluable tools for early intervention, personalized treatment plans, and proactive disease management.

## Performance evaluation of CNN in glaucoma diagnosis

*Assessment metrics for evaluating CNN performance*

The performance of CNN in glaucoma diagnosis can be evaluated using several assessment metrics. These metrics provide quantitative measures of the CNN’s effectiveness in classifying glaucomatous and healthy images. The following are commonly used assessment metrics: 

1. Accuracy. Accuracy measures the overall correctness of CNN’s predictions. It is calculated as the ratio of correctly classified images to the total number of images. A higher accuracy indicates a better classification performance [**33**].

2. Sensitivity. Also known as the true positive rate, sensitivity measures CNN’s ability to correctly identify glaucomatous images as positive. It is calculated as the ratio of true positives (correctly identified glaucomatous images) to the sum of true positives and false negatives (misclassified healthy images). A higher sensitivity indicates a higher rate of correctly identifying glaucomatous cases [**33**-**35**].

3. Specificity. Specificity measures CNN’s ability to correctly identify healthy images as negative. It is calculated as the ratio of true negatives (correctly identified healthy images) to the sum of true negatives and false positives (misclassified glaucomatous images). A higher specificity indicates a higher rate of correctly identifying healthy cases [**40**].

4. F1 score. The F1 score is a harmonic mean of precision and recall. It provides a balanced measure of the CNN’s accuracy, incorporating both sensitivity and specificity. The F1 score ranges from 0 to 1, with a higher score indicating better performance [**4**].

5. AUC-ROC. The area under the receiver operating characteristic curve (AUC-ROC) measures the CNN’s ability to distinguish between glaucomatous and healthy images across different classification thresholds. It plots the true positive rate against the false positive rate. A higher AUC-ROC indicates better discrimination performance [**36**].

*CNN performance in glaucoma diagnosis*

The use of CNNs in glaucoma diagnosis has shown promising results in achieving accurate and reliable classification of glaucomatous and healthy images. By leveraging deep learning algorithms and large datasets of labeled fundus images, CNNs have demonstrated their ability to automatically extract relevant features and detect subtle patterns associated with glaucoma [**32**]. The performance evaluation of CNN in glaucoma diagnosis involves assessing metrics such as accuracy, sensitivity, specificity, F1 score, and AUC-ROC. These metrics provide quantitative measures of CNN’s effectiveness in classifying images and distinguishing between glaucomatous and healthy cases. In comparison to traditional diagnostic methods, CNNs have shown superior performance, offering higher accuracy and specificity. The consistent use of reliable performance metrics is crucial in assessing the effectiveness of CNNs and their potential as valuable tools in glaucoma diagnosis.

In George et al.’s groundbreaking study, the effectiveness of optical coherence tomography (OCT) scans and retinal nerve fiber layer (RNFL) thickness measurements for glaucoma detection was demonstrated [**43**]. This study not only achieved impressive results in terms of high sensitivity and specificity in distinguishing between glaucoma patients and healthy individuals but also showcased the remarkable potential of OCT scans in providing detailed structural information about the retina. By utilizing OCT scans, George et al. took advantage of a non-invasive imaging technique that offers numerous benefits. Patients can undergo the procedure without discomfort or the need for invasive measures, leading to improved patient compliance and reduced risks associated with more invasive diagnostic approaches. This aspect alone makes the study highly advantageous and highlights its potential for widespread clinical implementation. Moreover, the use of RNFL thickness as a biomarker for glaucoma diagnosis is a key strength of this study. The ability to measure and analyze changes in the thickness of the retinal nerve fiber layer provides valuable insights into the progression of glaucoma and allows for the identification of early structural changes associated with the disease. This quantitative approach not only aids in accurate diagnosis but also enables monitoring of disease progression over time, providing clinicians with valuable information for effective management and treatment planning. The study’s merits extend beyond its methodology and results. By employing OCT scans and RNFL thickness measurements, George et al. addressed a critical need in the field of glaucoma detection, in which early and accurate diagnosis is crucial for preserving the patient’s vision. Their research contributes to the growing body of evidence supporting the use of non-invasive imaging techniques and quantitative measurements for glaucoma diagnosis, reinforcing the importance of incorporating these advancements into routine clinical practice.

In the study conducted by Diaz-Pinto et al., a compelling approach for glaucoma detection was employed by combining machine learning algorithms with statistical features extracted from fundus images [**44**]. The results of this study demonstrated good performance in terms of sensitivity, specificity, and accuracy, indicating its potential as an effective diagnostic tool. One notable advantage of this study is the utilization of fundus images, which offer a quick and non-invasive method for assessing the retina. Fundus imaging is a widely available and routinely used imaging technique in ophthalmic clinics, making it highly accessible for glaucoma screening and diagnosis. The simplicity and ease of obtaining fundus images enhance the practicality and feasibility of implementing this approach in various clinical settings. By extracting a range of statistical features from the fundus images, the model developed by Diaz-Pinto et al. successfully captured important characteristics of glaucomatous changes in the retinal vasculature and optic disc. These features served as valuable indicators of disease progression and helped differentiate between glaucoma patients and healthy individuals. The inclusion of such features enhances the robustness of the diagnostic model and contributes to its overall accuracy and performance. The merits of this study extend beyond the methodology itself. The combination of machine learning algorithms with fundus image analysis represents a significant advancement in the field of glaucoma detection. By harnessing the power of AI and statistical analysis, this approach offers a promising avenue for improving diagnostic accuracy and efficiency. The potential for widespread implementation in clinical settings is substantial, given the availability of fundus imaging technology and the ease of integrating machine learning algorithms into existing healthcare systems. 

Gheisari et al. employed advanced deep learning techniques, specifically CNNs and recurrent neural networks (RNNs), to tackle glaucoma detection using both spatial and temporal features extracted from retinal images and videos [**45**]. By integrating CNN and RNNs, the researchers achieved remarkable performance in terms of sensitivity, specificity, and F-measure, thereby establishing a new benchmark for accurate glaucoma diagnosis. One significant advantage of this study lies in its ability to capture dynamic changes in retinal videos. By incorporating temporal information through RNNs, the model was able to discern glaucoma-specific patterns and abnormalities that may not be evident in static retinal images alone. This innovative approach allows for the identification of subtle changes in the retinal vasculature, optic disc, and other relevant structures over time, enhancing the accuracy and sensitivity of the diagnostic process. Moreover, the utilization of state-of-the-art deep learning algorithms, such as CNN and RNNs, highlights the study’s bioengineering significance. Deep learning methods have revolutionized various fields, including medical imaging and diagnosis, by effectively leveraging the power of neural networks to extract complex features and patterns from large datasets. In the context of glaucoma detection, CNNs excel in capturing spatial features from retinal images, while RNNs excel in modeling temporal dependencies in retinal videos. The fusion of these two approaches enables a comprehensive analysis of both spatial and temporal features, leading to improved diagnostic accuracy. By employing deep learning techniques, Gheisari et al. have advanced the field of glaucoma detection by providing a robust and accurate methodology that surpasses traditional approaches. The integration of CNN and RNNs not only allows for the identification of glaucoma-specific patterns but also provides valuable insights into disease progression and response to treatment. The use of deep learning algorithms in glaucoma diagnosis holds immense potential for optimizing patient care, facilitating early detection, and enabling personalized treatment strategies. 

The complexity of the methodologies employed in the studies varies. George et al. focused on OCT scans and RNFL thickness measurements, which provide detailed structural information but may require specialized equipment and expertise for interpretation. Diaz-Pinto et al. utilized machine learning algorithms and statistical features extracted from fundus images, offering a simpler approach that can be easily implemented in clinical settings. Gheisari et al.’s study incorporated deep learning techniques, including CNN and RNNs, which are computationally intensive but have demonstrated exceptional performance in capturing glaucoma-specific patterns.

George et al. and Diaz-Pinto et al. utilized non-invasive imaging techniques - OCT scans and fundus images, respectively - to assess the retinal structures for glaucoma detection. These techniques are widely available and have been extensively used in clinical practice. Gheisari et al. employed retinal images and videos, capturing dynamic changes in the retina, thereby providing additional information beyond static images. This approach has the potential to enhance diagnostic accuracy by capturing temporal variations and subtle changes in the retinal features.

Gheisari et al. achieved the highest performance in terms of sensitivity, specificity, and F-measure by utilizing deep learning techniques and combining CNN and RNNs. This indicates that their model excelled in accurately distinguishing glaucoma patients from healthy individuals. Diaz-Pinto et al. demonstrated good performance in terms of sensitivity, specificity, and accuracy using machine learning algorithms and statistical features extracted from fundus images. George et al. also achieved high sensitivity and specificity using OCT scans and RNFL thickness measurements. While all studies demonstrated good performance, Gheisari et al. achieved the highest accuracy, making their approach particularly noteworthy. 

Diaz-Pinto et al.’s study using fundus images has the advantage of simplicity and potential for widespread implementation in clinical settings. Fundus cameras are widely available, making their approach easily accessible to healthcare providers. George et al.’s use of OCT scans, while non-invasive, may require specialized equipment and trained personnel. Although computationally intensive, Gheisari et al.’s deep learning approach offers the potential for automated and accurate glaucoma diagnosis once trained models are deployed. 

*CNN versus traditional diagnostic methods*

Zafar et al. demonstrated the superiority of CNNs over traditional methods in the context of glaucoma detection. Their study highlighted the potential of CNN in leveraging the vast amount of data collected from imaging tests commonly used in ophthalmology. By focusing on specific glaucoma scenarios, such as fundus photography screening and the diagnosis and detection of glaucoma progression through OCT imaging, they emphasized the applicability of CNN algorithms in these areas. One of the key advantages of CNN highlighted by Zafar et al. is their ability to analyze and classify images with performance that is equal to or even superior to human experts. 

With recent advances in computer technology and the availability of large datasets, CNNs have emerged as powerful tools for image classification and modeling. In the field of ophthalmology, in which extensive imaging data is available, CNN can effectively process and evaluate this data, providing accurate and efficient analysis. Zafar et al. also addressed the challenges associated with developing CNN models for glaucoma screening, diagnosis, and progress detection. They discussed the training and validation processes involved in building a CNN algorithm specifically tailored for glaucoma. This highlights the importance of training CNN on relevant datasets to ensure its accuracy and reliability in detecting glaucoma-related abnormalities.

Upon reviewing the study conducted by Ajitha et al., it is clear that their deep learning-based model for glaucoma diagnosis using retinal fundus images presents significant advantages over traditional diagnostic methods. The non-invasive and cost-effective nature of retinal fundus imaging makes it a practical and accessible technique for a larger population, potentially enhancing early detection and intervention for glaucoma. The automation provided by the deep learning model is particularly noteworthy, as it streamlines the analysis process and improves diagnostic efficiency. 

By automating the classification of retinal fundus images, the model enables faster and more accurate identification of glaucomatous and normal images, which can potentially alleviate the workload on ophthalmologists and healthcare providers. The model’s impressive performance in accurately distinguishing between glaucomatous and normal images further strengthens its potential clinical utility. With a specificity and precision of 100% in both softmax and SVM classifiers, the model demonstrates its ability to accurately identify normal images. Additionally, the high sensitivity, particularly in the SVM classifier, at 89.58%, highlights its proficiency in detecting glaucomatous images. 

Kucur et al. aimed to assess the effectiveness of a CNN classifier in discriminating between early-glaucoma and control visual fields (VFs) using multi-scale spatial information [**13**]. The researchers utilized two datasets and trained a custom-designed CNN using a novel Voronoi representation of the VFs. The results showed that the CNN achieved the highest average precision (AP) score across all test folds for one dataset and the third-best AP score for the other dataset. The CNN consistently outperformed other methods, including Mean Defect (MD), the square root of Loss Variance (sLV), and a Neural Network (NN) without convolutional features. The computed saliency maps provided valuable information on the regions of the VF contributing to the classification decision. The proposed CNN demonstrated excellent classification performance in distinguishing between early-glaucoma and control VFs compared to standard clinical decision measures. Supported by saliency visualization, CNN holds the potential to assist clinicians in automating the discrimination of early-glaucomatous and normal VFs. The findings emphasize the utility of CNN in advancing glaucoma detection and potentially improving patient care.

*Importance of consistency in CNN models*

Consistency plays a vital role in the development and application of CNN models for glaucoma. Consistency ensures that the CNN model produces reliable and reproducible results, which are essential for accurate glaucoma diagnosis, screening, and monitoring. Firstly, consistent CNN models enable consistent interpretation and decision-making. In the field of glaucoma, in which timely and accurate diagnoses are critical, consistency ensures that the CNN model consistently identifies and classifies glaucomatous abnormalities in retinal images. This reduces the risk of misdiagnosis or missed diagnoses, allowing healthcare professionals to provide appropriate treatment and intervention at the earliest stages of the disease [**4**].

The consistency in CNN models also promotes comparability across different studies and datasets. When multiple research studies or clinical trials utilize consistent CNN models, it becomes easier to compare their findings and evaluate the effectiveness of different algorithms, techniques, or datasets. This comparability enables the identification of robust patterns, trends, or biomarkers associated with glaucoma, leading to improved understanding and advancements in the field. Moreover, consistent CNN models enhance the generalizability of findings. Glaucoma datasets often vary in terms of demographics, imaging equipment, imaging protocols, and other factors. By developing CNN models that consistently perform well across diverse datasets, researchers can enhance the model’s ability to generalize its findings to real-world clinical settings. This increases the reliability and applicability of CNN models for glaucoma detection and diagnosis in different patient populations [**5**,**43**,**45**].

The consistency in CNN models also facilitates the integration of these models into clinical practice. When CNN models consistently demonstrate high performance, reproducibility, and reliability, they gain trust and acceptance from healthcare professionals. Consistent performance metrics enable clinicians to assess the CNN model’s performance over time, ensuring that it remains reliable and effective as new data and cases are encountered. Furthermore, consistent CNN models promote transparency and accountability. By maintaining consistency in the architecture, training process, and evaluation metrics, researchers can communicate the limitations, strengths, and biases of their models. This transparency fosters trust among healthcare professionals, patients, and regulatory authorities, encouraging responsible and ethical use of CNN models for glaucoma management [**32**-**34**].

## Advantages and limitations of CNN in glaucoma

CNNs have emerged as powerful tools in the field of glaucoma due to their ability to process and analyze large amounts of retinal image data. CNNs offer several advantages in the diagnosis, screening, and management of glaucoma.

*Advantages of CNN in terms of automation, efficiency, and objectivity*

CNNs have demonstrated several advantages in the context of glaucoma diagnosis, particularly in terms of automation, efficiency, and objectivity. These advantages contribute to improved accuracy and effectiveness in detecting glaucoma-related abnormalities. One major advantage of CNNs is their ability to automate the analysis of retinal images, specifically in the context of glaucoma screening and diagnosis. Traditional methods often require manual assessment by ophthalmologists, which can be time-consuming and subjective. On the other hand, CNN can automatically process and evaluate retinal images, enabling faster and more efficient diagnosis. This automation reduces the burden on healthcare providers and enhances the scalability of glaucoma screening programs [**33**,**34**].

Furthermore, CNNs offer improved efficiency in the diagnostic process. The automated nature of CNN algorithms allows for faster classification of retinal images, enabling prompt identification of potential glaucomatous abnormalities. This efficiency is particularly valuable in managing the large volumes of imaging data that ophthalmologists encounter in their clinical practice. Another advantage of CNNs is their objectivity in feature extraction and classification. Unlike traditional methods that rely on manual feature engineering, CNN can learn and extract relevant features directly from the retinal images. This objectivity reduces the potential for human error and variability in interpretation, leading to more consistent and reliable results. CNN can capture intricate patterns, textures, and spatial relationships in the retinal images, enhancing the sensitivity and specificity of glaucoma detection [**44**].

Several studies have demonstrated the advantages of CNN in glaucoma diagnosis. For example, Zafar et al. developed a CNN model specifically tailored for glaucoma screening and emphasized the automation and efficiency achieved through their approach [**46**]. Similarly, Ajitha et al. presented a deep learning-based model for glaucoma diagnosis using retinal fundus images, highlighting the objectivity and improved diagnostic accuracy offered by CNN [**47**].

*Limitations in implementing CNN in clinical practice*

While CNNs offer numerous advantages for glaucoma diagnosis, their implementation in clinical practice is not without limitations and challenges. It is crucial to be aware of these factors to ensure the appropriate utilization and interpretation of CNN models in glaucoma detection. One of the main limitations of CNN in clinical practice is the need for large and diverse datasets. Training CNN models requires a substantial amount of labeled data to effectively learn and generalize patterns. However, acquiring such datasets, especially in the context of glaucoma, can be challenging due to privacy concerns, limited availability, and the need for expert annotations. This limitation can potentially hinder the development and deployment of CNN models for glaucoma diagnosis [**41**,**42**].

Another challenge is the interpretability of CNN models. CNNs are often regarded as black-box models, meaning they provide accurate predictions, but lack transparency in explaining the underlying reasoning behind their decisions. In the field of healthcare, interpretability is crucial to build trust among clinicians and facilitate clinical decision-making. Addressing this challenge requires the development of explainable AI techniques that provide insights into the features and patterns utilized by CNN models to make predictions in glaucoma diagnosis. Furthermore, the integration of CNN models into existing clinical workflows and systems poses practical challenges. Adapting CNN algorithms to work seamlessly with different imaging modalities, electronic health record systems, and healthcare infrastructure can be complex and time-consuming. The successful implementation of CNN in clinical practice requires close collaboration between computer scientists, ophthalmologists, and healthcare professionals to ensure the integration is efficient, user-friendly, and compliant with regulatory standards [**4**,**28**,**32**,**34**,**45**].

Despite these limitations, research efforts are underway to address these challenges and enhance the clinical applicability of CNN in glaucoma diagnosis. For instance, Liu et al. proposed a method to generate synthetic glaucomatous images, augmenting the available dataset and potentially alleviating the data scarcity issue [**48**]. Additionally, efforts to develop explainable AI techniques, such as attention mechanisms and saliency mapping, are being explored to enhance the interpretability of CNN models in glaucoma diagnosis. Zheng et al. highlighted issues such as dataset bias, lack of diversity in training data, and the risk of overfitting in deep learning models [**49**]. These challenges emphasize the importance of addressing dataset limitations and ensuring the robustness and generalizability of CNN models in clinical practice.

*Addressing limitations and optimizing CNN for glaucoma diagnosis*

Addressing limitations and optimizing CNNs for glaucoma diagnosis is of paramount importance for several reasons. 

Firstly, by acknowledging and addressing the limitations of CNN, researchers, and clinicians can improve the accuracy and reliability of glaucoma diagnosis. CNNs are powerful tools for automated image analysis, but they are not without their shortcomings. Limitations such as dataset bias, insufficient diversity in training data, and overfitting can impact the generalizability and performance of CNN models [**43**]. By actively working to address these limitations, researchers can enhance the robustness of CNN and ensure their effectiveness in real-world clinical settings. Optimizing CNN for glaucoma diagnosis also enables the development of more efficient and scalable diagnostic systems. By improving the efficiency of CNN models, the diagnostic process can be streamlined, reducing the time and effort required for accurate glaucoma assessment. This is particularly crucial given the increasing prevalence of glaucoma and the need for timely diagnosis and treatment [**48**,**49**].

Furthermore, optimizing CNN can help address the challenges associated with limited resources and expertise in certain healthcare settings. For fine-tuning CNN models to perform well with limited data or by implementing transfer learning techniques, the diagnostic capabilities of these models can be extended to areas in which access to large, diverse datasets is limited. Lastly, addressing limitations and optimizing CNN fosters trust and acceptance among clinicians and healthcare providers. By demonstrating the reliability, reproducibility, and accuracy of CNN models, clinicians are more likely to adopt these technologies in their practice [**37**,**39**]. This can lead to improved patient care, earlier detection of glaucoma, and better management of the disease.

## Public health relevance and impact

By leveraging the power of deep learning and image analysis, CNNs have the potential to revolutionize the field of ophthalmology and significantly improve glaucoma diagnosis and management.

*The public health burden of glaucoma*

Glaucoma poses a significant public health burden globally, affecting millions of people and presenting substantial challenges to healthcare systems. The discussion on the public health burden of glaucoma is essential for understanding the magnitude of the problem and implementing effective strategies to mitigate its impact. 

Firstly, glaucoma is a leading cause of irreversible blindness worldwide [**1**,**2**]. According to the World Health Organization (WHO), it is estimated that over 70 million people are living with glaucoma, and this number is projected to increase significantly in the coming years due to aging populations and other risk factors [**50**]. The burden of glaucoma-related blindness not only affects individuals but also has a profound impact on their families, communities, and society as a whole. Secondly, the economic implications of glaucoma are substantial. The costs associated with glaucoma diagnosis, treatment, and long-term management are significant for both individuals and healthcare systems. These costs include expenses related to diagnostic tests, medications, surgical interventions, rehabilitation, and supportive care [**11**].

Moreover, the indirect costs due to productivity losses and reduced quality of life further contribute to the economic burden of glaucoma. Furthermore, glaucoma imposes a considerable social and psychological burden on individuals and their families. Vision loss or impairment can significantly impact one’s ability to perform daily activities, maintain employment, and participate fully in society [**51**]. The psychosocial consequences, including decreased independence, anxiety, depression, and social isolation, can have far-reaching effects on the well-being and quality of life of individuals with glaucoma [**52**].

Addressing the public health burden of glaucoma requires a comprehensive approach. This includes raising awareness about the importance of regular eye examinations and early detection, promoting access to affordable and high-quality eye care services, and implementing effective strategies for glaucoma prevention, diagnosis, and management.

*Role of CNN in improving access to accurate glaucoma diagnosis, particularly in underserved populations*

CNNs play a crucial role in improving access to accurate glaucoma diagnosis, especially in underserved populations. Underserved populations often face challenges in accessing specialized healthcare services, including ophthalmic care. This can result in delayed diagnosis, limited treatment options, and increased risk of vision loss. By leveraging CNN, we can address these disparities and bridge the gap in glaucoma diagnosis for underserved populations. One key advantage of CNNs is their ability to automate and standardize the diagnostic process. Traditional methods of glaucoma diagnosis require expertise from ophthalmologists and access to specialized equipment [**45**]. This poses a significant barrier in underserved areas in which resources and skilled professionals may be scarce. On the other hand, CNN can analyze retinal images and identify glaucoma-related abnormalities with high accuracy and objectivity. This automation eliminates the need for extensive manual interpretation and reduces the dependency on specialized human resources, making the glaucoma diagnosis more accessible. 

Furthermore, CNN can facilitate telemedicine and remote diagnosis, which is particularly valuable for underserved populations in rural or remote areas. Retinal images can be captured using fundus cameras and transmitted to centralized locations in which CNN models can analyze them. Ophthalmologists or trained technicians in these remote locations can then interpret the results and provide appropriate recommendations or referrals [**53**]. This telemedicine approach enables underserved populations to access expert opinions and accurate glaucoma diagnosis without the need for physical visits to distant healthcare facilities. In addition to improving access, CNN can also enhance the efficiency of glaucoma diagnosis. By automating image analysis, CNN can significantly reduce the time required for diagnosis [**54**]. This is crucial in underserved populations in which long waiting times and limited healthcare resources are common. The efficient utilization of CNN can help in the timely identification of glaucoma cases, enabling prompt interventions and management.

*Importance of integrating CNN-based diagnostic tools into public healthcare systems*

The integration of CNN-based diagnostic tools into public healthcare systems has immense importance in both general health and glaucoma care. CNNs have demonstrated remarkable capabilities in accurately and efficiently analyzing medical data, aiding in early detection, diagnosis, and treatment decision-making processes [**10**]. By incorporating CNN-based diagnostic tools into public healthcare systems, we can enhance patient outcomes, optimize resource utilization, and improve overall healthcare delivery. 

In general healthcare, CNN-based diagnostic tools can assist in the early detection and diagnosis of various medical conditions, ranging from cardiovascular diseases to skin disorders. The automated analysis of medical images by CNN models, such as X-rays, MRIs, and CT scans, can help healthcare professionals identify abnormalities and guide appropriate treatment strategies. This integration has the potential to reduce diagnostic errors, expedite the referral process, and ultimately improve patient prognosis [**33**].

In the context of glaucoma, the integration of CNN-based diagnostic tools into public healthcare systems can revolutionize the field of ophthalmology. CNN models trained on large datasets of retinal images can accurately identify glaucoma-related abnormalities, facilitating early intervention and preserving patients’ visual health [**28**]. By integrating these tools, healthcare systems can enhance glaucoma screening programs, improve accessibility to specialized care, and potentially reduce the burden on ophthalmologists.

*Potential cost-effectiveness and resource-saving benefits of CNN implementation in glaucoma screening programs*

The implementation of CNN in glaucoma screening programs has the potential to deliver significant cost-effectiveness and resource-saving benefits. Being a chronic eye condition leading to irreversible vision loss, glaucoma poses a substantial economic burden on healthcare systems worldwide [**55**]. By leveraging CNN technology, we can optimize the efficiency of glaucoma screening, reduce unnecessary referrals, and allocate resources more effectively. One key advantage of CNN implementation is its ability to automate the analysis of retinal images, which are commonly used in glaucoma diagnosis. Traditional screening methods often require manual interpretation by ophthalmologists, resulting in time-consuming processes and increased costs. CNN can swiftly process large volumes of images and accurately detect glaucoma-related abnormalities, enabling faster and more efficient screening. This automation not only saves time but also minimizes the need for specialized human resources, making it particularly valuable in regions with limited access to ophthalmologists [**56**].

Furthermore, CNN implementation can reduce healthcare costs associated with misdiagnosis and overtreatment of glaucoma. Misdiagnosing glaucoma can lead to unnecessary referrals, additional diagnostic tests, and inappropriate interventions, all of which contribute to higher healthcare expenditures. With their high accuracy in detecting glaucomatous changes, CNNs can aid in avoiding unnecessary procedures and interventions, ensuring that resources are directed toward patients who truly require specialized care [**57**]. Additionally, CNN implementation in glaucoma screening programs can lead to earlier detection and intervention, potentially preventing disease progression and reducing long-term treatment costs. The timely identification of glaucoma allows for the implementation of more cost-effective treatments and management strategies, minimizing the need for advanced interventions or surgeries in the later stages of the disease. By improving the efficiency and accuracy of screening, CNN can contribute to substantial cost savings over the long term. In summary, integrating CNN technology into glaucoma screening programs offers the potential for cost-effectiveness and resource-saving benefits [**58**]. The automation of image analysis, reduction in misdiagnosis, and earlier detection of glaucoma can optimize healthcare resource utilization, minimize unnecessary referrals and interventions, and ultimately lead to improved patient outcomes while alleviating the economic burden on healthcare systems.

## Future directions and emerging trends

*Key Considerations*

Focusing on enlarging and diversifying the datasets used for training CNN models is imperative. This includes incorporating data from various ethnic populations, different stages of glaucoma, and diverse imaging modalities. By utilizing comprehensive and representative datasets, CNN models can be trained to detect subtle variations and abnormalities associated with glaucoma across a broader range of patients. 

Transfer learning techniques need to be explored further to enhance the performance of CNN models in glaucoma diagnosis. By leveraging pre-trained models on large datasets from related tasks, researchers can optimize CNN architectures for glaucoma-specific features and reduce the need for extensive training on limited datasets. This approach holds promise for improving the generalizability and robustness of CNN models [**59**].

Addressing the “black box” nature of CNN models is an ongoing research focus. Efforts are being made to develop methods that provide explanations and interpretations for the decisions made by CNN models in glaucoma diagnosis. Explainable AI techniques aim to provide clinicians with transparent insights into the features and patterns that contribute to the model’s predictions, enhancing trust, and facilitating clinical decision-making [**60**].

Seamlessly integrating CNN-based diagnostic tools into the clinical workflow to ensure practical and user-friendly implementation is the ultimate endpoint to be achieved [**55**]. This involves developing user interfaces and software platforms that enable efficient image acquisition, automated analysis, and integration of CNN results with electronic health records. These developments aim to facilitate the adoption and integration of CNN models into routine clinical practice by streamlining the diagnostic process. 

Developing CNN models that can provide real-time analysis and immediate feedback at the point of care is of paramount importance in terms of public health relevance. This includes the exploration of hardware-accelerated CNN implementations and the development of portable or handheld imaging devices that can capture and process retinal images in real time. These advancements aim to extend the reach of CNN-based glaucoma diagnosis to underserved areas and enable prompt diagnosis in urgent clinical settings.

*Significance of continued research and innovation in CNN and glaucoma*

The importance of continued research and innovation in the field of CNN for glaucoma management cannot be overstated. As technology advances and new methodologies emerge, it is crucial to continuously explore and refine CNN models to enhance their accuracy, efficiency, and applicability in diagnosing and managing glaucoma. Firstly, glaucoma is a complex and multifactorial disease, and there is still much to be understood about its pathophysiology and progression. By conducting further research, scientists and clinicians can gather more comprehensive datasets and identify novel biomarkers or imaging techniques that can be incorporated into CNN models. This ongoing research can contribute to the development of more precise and sensitive algorithms, enabling the earlier detection and intervention of glaucoma cases. Moreover, glaucoma management extends beyond diagnosis and includes monitoring disease progression, treatment response, and long-term outcomes. Continued research in CNN can help refine the predictive capabilities of these models, enabling more accurate prognostication and personalized treatment planning for glaucoma patients. Additionally, incorporating real-time monitoring and analysis of clinical data into CNN models can facilitate timely interventions and improve patient outcomes. Furthermore, the field of CNN is continuously evolving, and new advancements, such as transfer learning, ensemble models, and explainable AI techniques, hold promise for improving the performance and interpretability of CNN models in glaucoma management. Investing in research and innovation in these areas can lead to breakthroughs in diagnostic accuracy, clinical decision support, and patient care.

## Conclusion

In conclusion, the integration of CNNs in glaucoma detection represents a paradigm shift in ophthalmology, offering significant advancements in accuracy, efficiency, and accessibility. The studies reviewed in this paper demonstrate the potential of CNN models to revolutionize glaucoma diagnosis and screening. CNNs have shown remarkable performance in automatically extracting relevant features and detecting subtle patterns associated with glaucoma. By leveraging deep learning algorithms and large datasets of labeled fundus images, OCT scans, and retinal videos, CNN models have achieved high sensitivity, specificity, and accuracy in distinguishing between glaucoma patients and healthy individuals. This showcases the potential of CNN to improve early detection, facilitate clinical decision-making, and enhance patient outcomes. However, the implementation of CNN models in clinical practice comes with challenges that need to be addressed. These include data scarcity, interpretability, dataset biases, and integration into existing clinical workflows. Efforts are being made to overcome these challenges by leveraging transfer learning techniques, developing explainable AI techniques, and optimizing data collection strategies. By addressing these limitations, CNN models can be optimized for glaucoma diagnosis, improving their reliability, robustness, and generalizability. This advancement not only holds promise for improving the detection and management of glaucoma but also has the potential to alleviate the burden on healthcare systems and enhance access to quality eye care, particularly for underserved populations. Overall, the integration of CNN in glaucoma detection represents a transformative approach that has the potential to revolutionize ophthalmology. Continued research, collaboration, and innovation in this field will contribute to further advancements, ultimately benefiting patients, clinicians, and healthcare systems worldwide.

**Conflict of Interest statement**

None of the authors has any conflict of interest to disclose.

**Acknowledgments**

None.

**Sources of Funding**

No funding was received.

**Disclosures**

None.
